# Editorial

**Published:** 1891-10

**Authors:** 


### Editorial.

### THE GRADY HOSPITAL.

The Grady Hospital of this city is now resting in the hands of the city and its citizens as to whether or not its completion is to be a thing of the future. All have contributed liberally, and Atlanta's nobly remembered citizen, Mr. W. A. Moore, has added to the perpetuity of his memory by a gift of $7,500 to the Hospital at the time of his death. But the Hospital is not completed, nor is it nearing it. Had it been a less extensive and elaborate affair, and a building simply to meet the present demands of the population, it would have by this time been completed and rendering the service for which it was intended, and as the city grew, if there was an increased demand for hospital accommodation, another could have been erected in the opposite portion of the city. and thus give equal opportunities to all portions. The elaborate manner in which the buildings are extended over a broad expanse would meet the requirements of a city twice its size, but they are attractive and commodious and we will lend all of our efforts for its success.

### SOUTHERN ASSOCIATIONS.

We note with pleasure the appearance of the Opthalmic Record upon the arena of journalism. It is published in Nashville, and having such a man as Dr. G. C. Savage at its head insures its success. Opthalmic journals have been confined too exclusively to the North and West and the appearance of this new Southern journal supplies a long felt want where Southern men engaged in this department of medical work can give expression to their views. We wish it success. Apropos to the last issue Dr. Savage has started the discussion of a subject which will have our hearty support.

It is the establishment of the Mississippi Valley Opthalmological Association. Why not? The time is ripe for its birth, for this branch of our medicine is upheld by many distinguished men in this Southern clime, and their uniting themselves together into an Association will foster and advance this now growing specialty. The Southern Surgical and Gynæcological Association which only a few years ago saw the light of day, has done more toward inspiring zeal into our profession and advancing this special line of work than everything put together in the last thirty years. Let the good work go on.

---

### THE DOCTOR'S BILL.

---

Our present legislature is in many respects a remarkable body of men. Some of them must have a secret Delphian Oracle from which they imbibe those acts of wisdom which have eternally stamped them as "makers of their own destiny." And to think that some who profess to belong to our own fraternity should be guilty of these "eccentric flights." But "in the midst of life we are in death." Surely the creative mind of some is unlimited in its bounds. The Doctor's Bill which has lately passed both houses of the Legislature is affording much amusement to our editors of the various medical journals, as is exemplified in their satirical criticisms of this body of distinguished Georgians. The bill provides that any doctor who shall be found intoxicated when in the discharge of his professional duties shall be fined not less than $100 nor more than $300 for the first offence, and for the second he shall be fined not less than $300 nor more than $1,000 by the judge.

Brethren, take care! When a man has entered upon the practice of his profession he is just as much in the discharge of his professional duties when he is sitting in his office quitely waiting for patients as when he is attending at the bedside the sufferings of some disease-stricken invalid. And what patient who had enough confidence in a doctor to send for him to wait upon some member of his family would be base enough to go and report to the officer of the law that he had found this physician intoxicated? It is a well known fact that some of the brightest men in our profession have been addicted to the inordinate use of drink, and while this is not said to exonerate such from this pernicious habit, we have all seen instances where patients preferred to have their "own doctor" even though under the influence of liquor, than to have the services of some one else. Some one has said that drinking is more common among the medical profession than any other. Not granting the truth of the statement until further inquiry we must say that the life of a physician has certainly many weak places which will aid the furtherance of any habit which he has for drink. Night work, loss of sleep, weariness of the body and of the mind, often makes him feel as if a stimulant is necessary. But do not misconstrue our words in thinking that we advocate that for a physician to drink is justifiable. Far from it, for if there are any class of men who need a *sana mens in sano corpore* it is the men of the medical profession. But the idea of trespassing upon the personal rights of physicians even by a law the very enactment of which has the word nullity stamped upon its face, is preposterous.

Dr. Shrady, under the editorial of the N. Y. Medical Record of Sept. 19th, very plainly and truthfully remarks in regard to this new law: "The penalty imposed by the Georgians upon a drunken physician may perhaps not be too severe, but the passage of such a law by the Legislature is in the nature of a deliberate insult to the physicians of that State."

### FUTURE OF MARRIAGE.

The pessimistic view that the future of marriage is one of decline is very tersely handled by Mr. Jno. L. Heaton in the July number of the North American Review. This "neurasthenic age" of ours, as some would term it, is fast on the decline, and the above writer well says "it is becoming the fashion to be healthy. Walking, the gymnasium, horseback riding, bowling, fencing, are resorted to, and teachers of calisthenics, Swedish or Delsartean exercises, flourish by the hundred where ten years ago they starved by dozens. Dyspepsia is no longer the national disease. * * * * Exercise and outdoor air will drive away half the megrims which assault women, and with the corset will disappear a good proportion of the rest. It is only a few days ago that one of the daily papers informed us of the crusade being made against the wearing of corsets by a fervid Methodist evangelist in the province of Canada. The report tells us that so intense was the feeling at one of the evening meetings when the evangelist condemned the wearing of these articles of apparel that one woman tearing her garments from snow-white shoulders, unfastened her corset with a demoniacal look and flung it into the fire outside of the tent. This was followed instantaneously by all the women in the meeting, and a sight unparalleled in history revealed itself as the flames lit up this mass of burning corsets. Surely this must be an age of frenzy. Even the New York show girls intend to abandon long skirts on wet days and to wear petite knee skirts and high boots to hide their dainty limbs. The writer says that not even does the entrance of women into professions long considered only suitable for men, preclude the idea of a diminution in marriages for, says he, "doctors of unlike sex marry and divide the patients, the profits and the works." But does not the professional work of a woman end when she marries a man capable of giving her the support for which she was laboring? The concluding remarks of the writer are terse indeed: "So long as grass grows green and water runs downward to the sea will men and women share their joys and sorrows, cherish their offspring and build in happy hope the fabric of their homes. * * * * The rosy lips of Cupid utter an everlasting no to the cry that marriage is a failure."

### THE FUTURE OF MOSQUITOES.

Drs. Finlay and Delgado, of Havana, have been testing the properties of the attenuated virus of yellow fever by causing new arrivals to be subjected to the bites of those mosquitoes which had previously fed upon the body of patients suffering with that disease. So far the results of these inocculations have given some favorable signs of success. Thus we see that use can be made of the most ill-welcomed visitors. Everything was created for some purpose, and now that a new use has been made of this festive singer, his admirers will be contending with each other as to which is his most useful function in society, the driving of men away from malarial districts or inocculating of patients with the attenuated virus of yellow fever.

The Atlanta Society of Medicine has again resumed its meetings for the winter season. Its first meeting Tuesday night, Sept. 1st, was more of a reunion, the time being occupied in the report of cases.

On Tuesday night, Sept 15th, the Society enjoyed a paper read by Dr. Kime, entitled Physician vs. Gonorrhœa. The subject was well handled, as well as were the discussions subsequent. The Society of Medicine should be a pride to every physician in Atlanta, and they all should make it what it ought to be—an honor to the profession and a recognized medical illuminating centre to the whole country. It should be with no small degree of pride that writers for our medical journals can sign themselves, "Member of the Atlanta Society of Medicine." It can be done, and why not do it. Pluck, energy and talent are a characteristic of the Atlanta people, and surely the medical profession is not to be a body distinctive for its lethargy. Let us work with a vim and see that all the profession are interested in the same.

---

The Woman's Medical College of Georgia opens October 1st, with prospects of a large class. The ladies of the Board of Trustees, with Mrs. Governor Northen as President, and the Faculty, with Dr. A. G. Thomas as President and Dr. J. W. Stone as Dean, are bringing this institution to great usefulness to our Southern ladies. One-half reduction of lecture fees is granted to wives and daughters of physicians, clergymen and Confederate Veterans. For particulars, address Woman's Medical College, Atlanta, Ga., Box [illegible]15.

---

MASSILLON, OHIO, Sept. 14th, 1891.

ANTIKAMNIA CHEMICAL CO., St. Louis, Mo. :

Gentlemen :—"In the characteristic and excruciating frontal headache accompanying influenza, I immediately prescribe Antikamnia in five or six grain doses, repeated once in one or two hours, with the happiest results. I have learned to look upon it as almost a specific in counteracting this form of pain."

The above is an extract from an article on Influenza read by me before the "Stark County Academy of Medicine," at Canton, O., Sept. 1st., 1891. Very respectfully,

D. S. GARDNER, M. D.

